# Role of early post-operative breast MRI: how helpful is it in deciding the next step for women who may have residual disease?

**DOI:** 10.1259/bjro.20210024

**Published:** 2021-07-29

**Authors:** Nuala A Healy, John R Benson, Ruchi Sinnatamby

**Affiliations:** 1 Cambridge Breast Unit, Cambridge University Hospitals NHS Foundation Trust, Addenbrookes’ Hospital, Hills Road, Cambridge, UK

## Abstract

**Objectives:**

Positive resection margins following breast conserving surgery are a risk factor for local disease recurrence. Subsequent management of patients is often not straightforward, with post-operative breast MRI increasingly used to aid decision-making. Interpretation of MRI after surgery can prove challenging due to local inflammatory enhancement. We reviewed our experience of post-operative breast MRIs to determine their ability to detect residual disease and to evaluate how they changed initial patient management from re-excision to an alternative.

**Methods:**

A search of breast MRIs performed from August 2014 to December 2019 was undertaken, to identify those performed post-operatively within 4 months of breast conserving surgery. Electronic patient records and imaging were evaluated to determine additional work-up, pathology and surgical outcomes.

**Results:**

Of the 2274 breast MRIs during the study period, 44 (2%) were performed post-operatively to evaluate 47 breasts. MRI was normal in 20 cases (43%), suspicious findings at surgical cavity only in 13 (28%), suspicious ipsilateral distant breast findings only in 6 (13%), and both cavity and distant findings in 7 cases (15%). Contralateral abnormalities were identified in 3 cases. Following MRI, mastectomy was performed in 11 cases, re-excision in 25, with 2 subsequent mastectomies, and multidisciplinary team accepted margins in 11 cases, 10 of whom underwent post-operative radiotherapy. MRI altered initial patient management from re-excision to an alternative in 25 cases (45%).

**Conclusion:**

Post-operative breast MRI, although potentially challenging to interpret, can prove useful in planning the next step in patient management, particularly in its ability to evaluate the whole breast.

**Advances in knowledge:**

Post-operative breast MRI is increasingly requested at multidisciplinary team following breast conserving surgery with positive surgical margins on histology, however interpretation is challenging. The value of these studies lie in assessment of the distant breast rather than the surgical resection cavity and can alter patient management guiding the most appropriate next step for definitive treatment.

## Introduction

For many patients with early breast cancer, the surgical treatment options of breast conserving surgery (BCS) in conjunction with radiation therapy, or mastectomy can be equally effective, with no difference observed in disease-free and overall survival.^1,2^ Drivers towards preferentially choosing BCS include a smaller tumour volume to breast-size ratio, peripheral location, absence of multicentricity and patient choice.

Following BCS, a positive surgical resection margin is a risk for local disease recurrence and usually necessitates further surgery.^3–5^ For patients with invasive breast cancer that undergo BCS, international guidelines define clear or adequate surgical resection margins as “no tumour on ink”, although the Association of Breast Surgery (ABS) have decreed a minimum margin of 1mm.^6–8^ For ductal carcinoma *in situ* (DCIS), a more stringent mandate of either 1 mm (ABS) or 2 mm is appropriate and results in lower local recurrence rates.^8–11^ For patients with positive surgical resection margins, further surgical treatment options range from re-excision of the surgical cavity to mastectomy; whilst radiation therapy alone may suffice for those with “close” margins but no evidence of further disease.

Clinical decision-making for further surgery, and whether to recommend re-excision or mastectomy, is not always straightforward and can generate much debate at post-operative multidisciplinary team (MDT) meetings. The authors’ institution has increasingly accepted selective use of post-operative MRI as part of routine clinical service, primarily at the request of surgeons, to aid decision-making for patients with positive margins after initial BCS. Pre-operative breast MRI is widely used for local staging to determine tumour extent and guide further management. However, use of post-operative breast MRI to detect residual tumour can prove challenging due to breast parenchymal alterations and inflammatory post-surgical enhancement.^12,13^ The European Society of breast imaging (EUSOBI) guidelines state that breast MRI may be used following BCS, in three instances; firstly as a follow-up screening tool, secondly, to assess for local disease recurrence, and finally to detect residual disease in the early post-operative period.^14^ These guidelines therefore endorse early post-operative MRI whilst recommending that it should not subvert any indication for re-excision based on histologically positive margins.

The aim of this study was to review all breast MRIs performed post-BCS at a single institution over a period of 5 years, and determine their sensitivity for detection of residual disease either at the surgical or more distant sites in the ipsilateral breast. A secondary objective was to evaluate how post-operative breast MRI influenced patient management and led to any change from breast re-excision to an alternative.

## Methods and materials

### Patient selection

Multidisciplinary team (MDT) referral for selective use of early post-operative MRI is an accepted management pathway within our institution (Cambridge Breast Unit). This study was registered as a service evaluation with approval from the Quality and Safety Information System Clinical Audit team. As this was an observational study without intervention, formal ethics approval and individual patient consent was waived.

### Study population

A list of all breast MRIs performed from August 2015 to December 2019 was retrieved from the hospital radiology information system (RIS). Reports were reviewed to determine those MRIs performed early following BCS. Referrals for post-operative breast MRI were all made after MDT review of pre-operative imaging and wide local excision (WLE) specimen histology. Inclusion criteria were screened and symptomatic females of all ages who underwent breast MRI within 4 months of initial BCS to assess for residual disease. Positive margins were defined as tumour at ink and close margins as <1 mm for both invasive and *in-situ* disease as per ABS guidelines.^8^ Patients who had WLE for recurrence of previous breast cancer and patients who had MRI performed more than 4 months after their original surgery were excluded.

### Breast MRI technique

Breast MRIs were performed on an Optima MR450w 1.5 Tesla MRI scanner (GE Healthcare Chicago, Illanois) with dedicated 8-Channel phased array breast coil. Following acquisition of the localizer, a 2 mm axial *T*_2_ weighted sequence was obtained. *T*_1_ weighted three-dimensional (3D) fat-saturated (FS) gradient-echo images were initially acquired followed by administration of contrast medium (0.1 mmol/kg Gadobutrol (Gadovist)) at 2 ml s^−1^ administered via pump injector followed by a 20 ml saline flush) and dynamic high-resolution *T*_1_ weighted 3D FS gradient-echo images obtained. Five dynamic post-contrast enhancement phases were acquired with an acquisition time of 84 sec for each, all with 2 mm section thickness. Post-contrast subtracted images and post-processing MIP images were generated for review.

### Image interpretation

MRI images were reported by at least two experienced breast radiologists and agreed by consensus with a panel of breast radiologists, in accordance with our departmental protocol. All images were reviewed with evaluation of lesion morphology and post-contrast enhancement patterns with their associated enhancement kinetics determined using CADstream (Merge Healthcare Inc.). Distant disease was defined as disease distant from the rim-enhancing surgical resection cavity, which may be in the same quadrant but at least 1 cm away from the surgical cavity. Results of post-operative breast MRIs were discussed at breast MDT to agree further patient management. In cases where MDT advised second look ultrasound and where ultrasound detected an indeterminate lesion, a 14G core biopsy was performed under ultrasound guidance. If not visible on ultrasound and MDT recommendation was for biopsy prior to surgery 11G vacuum-assisted biopsy (VAB) was performed under MRI guidance. All biopsies were discussed at a subsequent multidisciplinary meeting.

To facilitate the study, a prospective, password protected database was constructed and maintained in Microsoft Excel. Electronic hospital records and patient archiving and communications systems (PACS) for each of the females were reviewed to establish patient demographics, additional breast imaging undertaken at assessment, biopsies performed, pathology findings and eventual patient outcomes.

## Results

A total of 2274 breast MRIs were performed between August 2014 and December 2019 in 1463 women. Breast MRI was performed within 4 months of BCS in 44 women (2% of breast MRIs performed). 41 females had unilateral and 3 women had bilateral BCS yielding a total of 47 breasts for evaluation. Over this period, a total of 1365 BCS procedures were undertaken indicating that post-operative breast MRI to detect residual disease was performed in 3% of BCS cases (47/1365). During this same period, the rate of re-operation for margin positivity following BCS at Cambridge Breast Unit was 17%.

On review of records, prior to initial surgery none of the females in our study met standard MDT criteria for, or had, breast MRI performed pre-operatively. Criteria for pre-operative MRI in our institution are guideline endorsed and include females with lobular cancer, mammographically occult cancer, multifocal and/or multicentric disease where BCS is being considered, females with increased risk and patients undergoing neoadjuvant systemic therapy.^12,14,15^ During the same period, 840 women underwent breast MRI for local staging prior to surgery. The average age of patients in this study was 56.5 years (range 40–73 years). The initial tumours were screen detected in 24 women; 20 presented symptomatically. The average length of time from initial BCS until post-operative breast MRI was 62.5 days (range 13–112 days). Table 1 details the demographics of the study population.

**Table 1.** Baseline demographics

Parameter	Number (%)
**Average patient age**	56.5 years with range 40–73 years
**Presentation Type:** **Screening**	24 (55%)
**Symptomatic**	20 (45%)
	
**Tumour type**	*N* = 47
**Invasive NST**	31 (66%)
**Invasive lobular**	3 (6%)
**Mixed ductal and lobular**	3 (6%)
**Metaplastic**	2 (4%)
**Mucinous**	1 (2%)
**Tubular**	1 (2%)
**High-grade DCIS**	6 (13%)
	
**Invasive tumour grade**	*N* = 41
**1**	3 (7%)
**2**	24 (59%)
**3**	14 (34%)
	
**Oestrogen receptor status**	*N* = 41
**Positive**	35 (85%)
**Negative**	6 (15%)
	
**HER2 receptor status**	*N* = 41
**Positive**	8 (20%)
**Negative**	33 (80%)
	
**Mean tumour size**	29 mm (Range 1–65 mm)
	
**Surgical management following post-operative breast MRI**	*N* = 47
**Mastectomy**	11
**Re-excision**	25
**Mastectomy after re-excision**	2
**No surgery**	11

DCIS, ductal carcinoma *in situ*.

### Indications for post-operative breast MRI

In 43 breasts, MRI was performed to evaluate for additional ipsilateral disease as margins were positive on histology, with *in-situ* or invasive disease. One female with bilateral disease had positive margins in one breast (comprising 1 of the 43 above) and close margins in the other. In a further case, MRI was performed due to lack of concordance between the tumour seen in the sentinel node and that in the BCS specimen, raising the possibility of additional occult disease. In another case, extensive pleomorphic lobular carcinoma in situ (LCIS) was present in an excision shave taken at time of BCS and in the final, 47th case, although margins were clear, MRI was performed to search for additional disease as there was discordance of tumour size between histology and conventional imaging. In 11 cases, DCIS was present at the positive margins which was non-calcified on conventional imaging.

### Surgical management following post-operative breast MRI

Following MDT review of post-operative breast MRI, further surgery was performed in 36 breasts with 11 proceeding directly to mastectomy and 25 undergoing re-excision of positive margins. It is worth noting that while the MDT may agree that re-excision rather than mastectomy would be appropriate, it is the conversation between the surgeon and the patient that ultimately governs the final surgical decision. Of these 25 re-excisions, 2 required subsequent completion mastectomy, giving 13 mastectomies in total. There were 11 cases for which the MDT agreed that no further surgery was required despite histologically positive margins. For context, in the same period, amongst all margin positive patients approximately, 75% underwent further surgery with an MDT endorsed decision for no further surgery in 25%. All females managed with BCS received post-operative breast irradiation except one with a relative contraindication of alpha-1 antitrypsin deficiency. Table 2 illustrates each of the 47 breasts that underwent post-operative breast MRI, their surgical outcomes and histology of the final surgery.

**Table 2.** Surgical outcomes and final histology for each of the 47 breasts that underwent post-operative breast MRI

Breast	Abnormal enhancement on MRI	Chemotherapy prior to further surgery	Surgical Outcome	Pathology post MRI
1	Cavity alone	NA	No further surgery	NA
2	Cavity alone	No	Mastectomy	1 mm lobular carcinoma and residual extensive pleomorphic LCIS
3	Cavity alone	No	Re-excision	2 mm residual HG DCIS
4	Cavity alone	No	Re-excision	No residual disease
5	Cavity alone	NA	No further surgery	NA
6	Cavity alone	No	Re-excision	2.5 mm HG DCIS
7	Cavity alone	No	Re-excision and mastectomy	Re-excision – 2 mm invasive NST and 24 mm HG DCIS Mastectomy - 4 mm focal residual intermediate and HG DCIS
8	Cavity alone	Yes	Re-excision	5mm G1 invasive carcinoma NST with residual high-grade DCIS with negative shaves.
9	Cavity alone	No	Re-excision	4 mm tubular carcinoma.
10	Cavity alone	No	Re-excision	No residual disease
11	Cavity alone	No	Re-excision	No residual disease
12	Cavity alone	No	Re-excision	No residual disease
13	Cavity alone	Yes	Re-excision	No residual disease
14	Distant alone	No	Re-excision	No residual disease
15	Distant alone	No	No further surgery	NA
16	Distant alone	No	Mastectomy	No residual disease
17	Distant alone	No	No further surgery	NA
18	Distant alone	Yes	Re-excision	No residual disease
19	Distant alone	No	Mastectomy	80mm HG DCIS, 80mm with 2mm focus of invasive carcinoma NST
20	Cavity and Distant	Yes	Mastectomy	14 mm HG DCIS
21	Cavity and Distant	Yes	Mastectomy	7 mm invasive carcinoma NST, 2 mm focus of intermediate-grade DCIS
22	Cavity and Distant	No	Re-excision and mastectomy	3 mm invasive carcinoma NST with extensive residual HG DCIS surrounding previous cavity site.
23	Cavity and Distant	Yes	Mastectomy	Multiple foci of residual invasive carcinoma NST (largest deposit 11 mm) and 81 mm extensive HG DCIS.
24	Cavity and Distant	Yes	Mastectomy	No residual disease
25	Cavity and Distant	No	Re-excision	No residual disease
26	Cavity and Distant	No	Mastectomy	7 mm Invasive carcinoma NST with associated HG DCIS
27	Contralateral breast only	No	Mastectomy	30 mm HG DCIS (non-calcified)
28	No	Yes	Re-excision	Classical LCIS but no invasive disease
29	No	No	Re-excision	No residual disease
30	No	No	Re-excision	Focal HG DCIS < 5 mm in total
31	No	No	Re-excision	No residual disease
32	No	No	Re-excision	No residual disease
33	No	No	Re-excision	No residual disease
34	No	No	Re-excision	No residual disease
35	No	No	Re-excision	Classical LCIS but no invasive disease
36	No	No	Re-excision	No residual disease
37	No	Yes	Re-excision	No residual disease
38	No	No	Re-excision	No residual disease
39	No	No	Mastectomy	2 mm Intermediate DCIS
40	No	Yes	Mastectomy	Two 4 mm foci of residual invasive carcinoma NST with focal DCIS.
41	No	NA	No further surgery	NA
42	No	NA	No further surgery	NA
43	No	NA	No further surgery	NA
44	No	NA	No further surgery	NA
45	No	NA	No further surgery	NA
46	No	NA	No further surgery	NA
47	No	NA	No further surgery	NA

DCIS, ductal carcinoma *in situ*; LCIS, lobular carcinoma in situ.

### Breast MRI results

**Figure 1.**
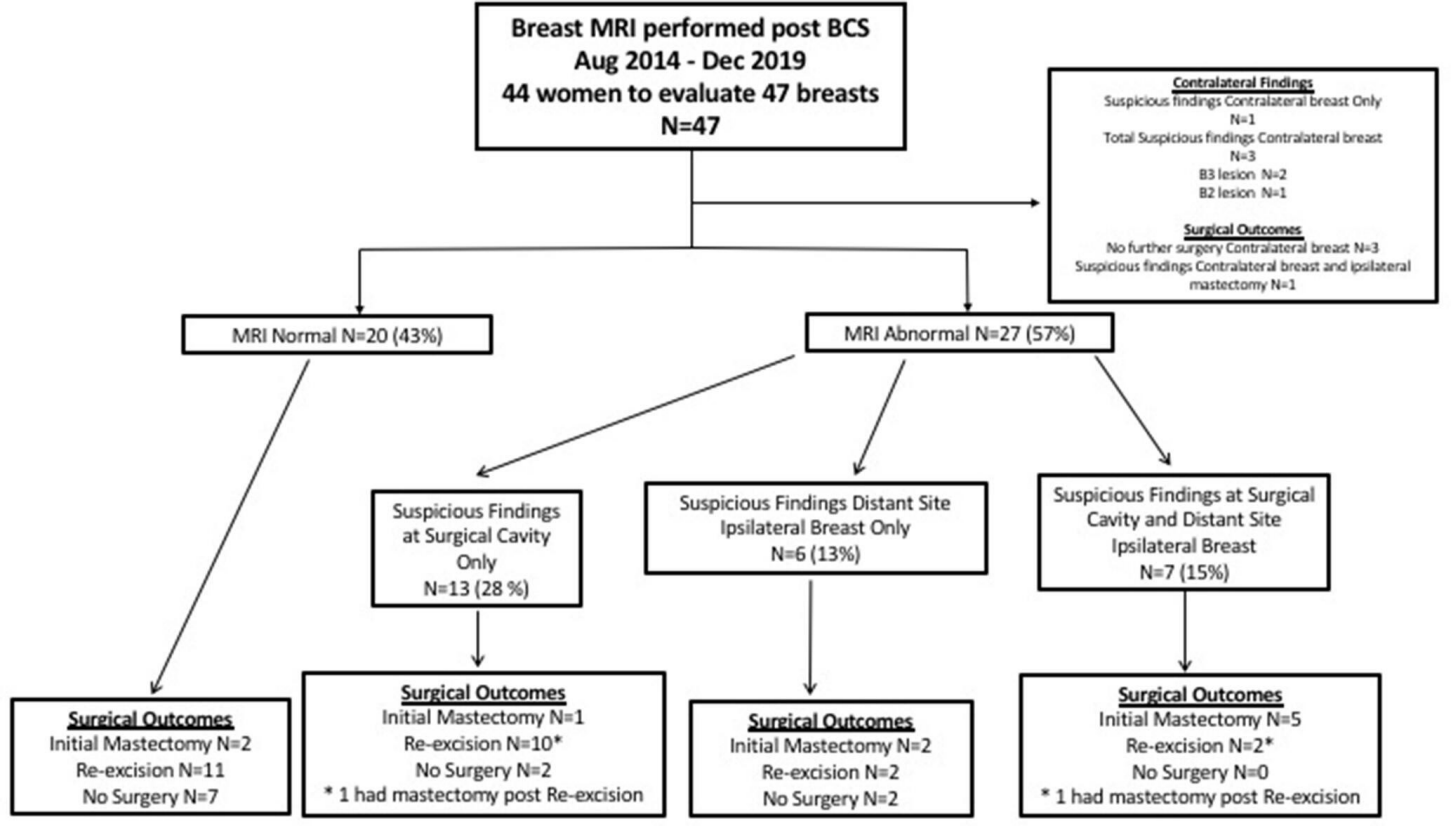
Flowchart demonstrating outcomes of females undergoing early postoperative breast MRI. BCS, breast conserving surgery.

Breast MRI was deemed non-suspicious for residual disease in 20 breasts (43%) with expected post-surgical changes but no additional areas of concerning enhancement in either breast. Abnormal enhancement on breast MRI, suspicious for residual disease at the surgical resection cavity, disease distant from the surgical cavity in the ipsilateral breast or contralateral abnormality was present in 27 breasts (57%). Figure 1 summarises the MRI results and outcomes.

### Contralateral abnormality on breast MRI

In three cases (7%), post-operative MRI identified abnormal enhancement in the contralateral breast. Second look ultrasound was normal in each, and all subsequently underwent MRI-guided breast biopsy. Final histology was classified as B3 for two lesions and benign (B2) in the third. The B3 lesions underwent percutaneous vacuum-assisted excision with no pathological upgrade and no further requirement for surgery. Nonetheless, in two of these cases, there was concomitant abnormal enhancement in the ipsilateral operated breast, at both the surgical cavity and distant to this (see below).

### Breast MRI abnormality distant to surgical cavity only

Abnormal enhancement distant from the site of surgery, with no suspicious enhancement in the vicinity of the surgical cavity, was observed in six breasts (13%). Amongst these cases, mastectomy was performed in two. In one, there was extensive residual disease. In the second, a 6 mm lesion on MRI was proven to be high-grade DCIS on VAB which was fully excised at that VAB with no further residual disease.

Re-excision was performed in two cases. In one of these there was enhancement 12 mm medial to the surgical resection cavity and core biopsy revealed classic LCIS (B3). Final histology following re- excision confirmed further LCIS but no additional upgrade. The patient has had 4 years of follow up with no recurrence of disease. The second case had 20 mm of abnormal enhancement, 16 mm anterior to the surgical resection cavity. She underwent chemotherapy prior to completion surgery with no residual disease detected at final surgery.

In the final two cases, no further surgery was performed. In the latter scenario, in one case, margins were accepted at MDT with a fully excised mucinous tumour and small foci of associated DCIS <1 mm from the inferior margin. Second look ultrasound of the area of concern on MRI confirmed appearances of fat necrosis. In the second case, margins were histologically negative but a size discordance between initial imaging and final pathology prompted MRI to seek additional occult disease. Second look ultrasound in this case confirmed the MRI finding as a normal intramammary lymph node.

**Figure 2.**
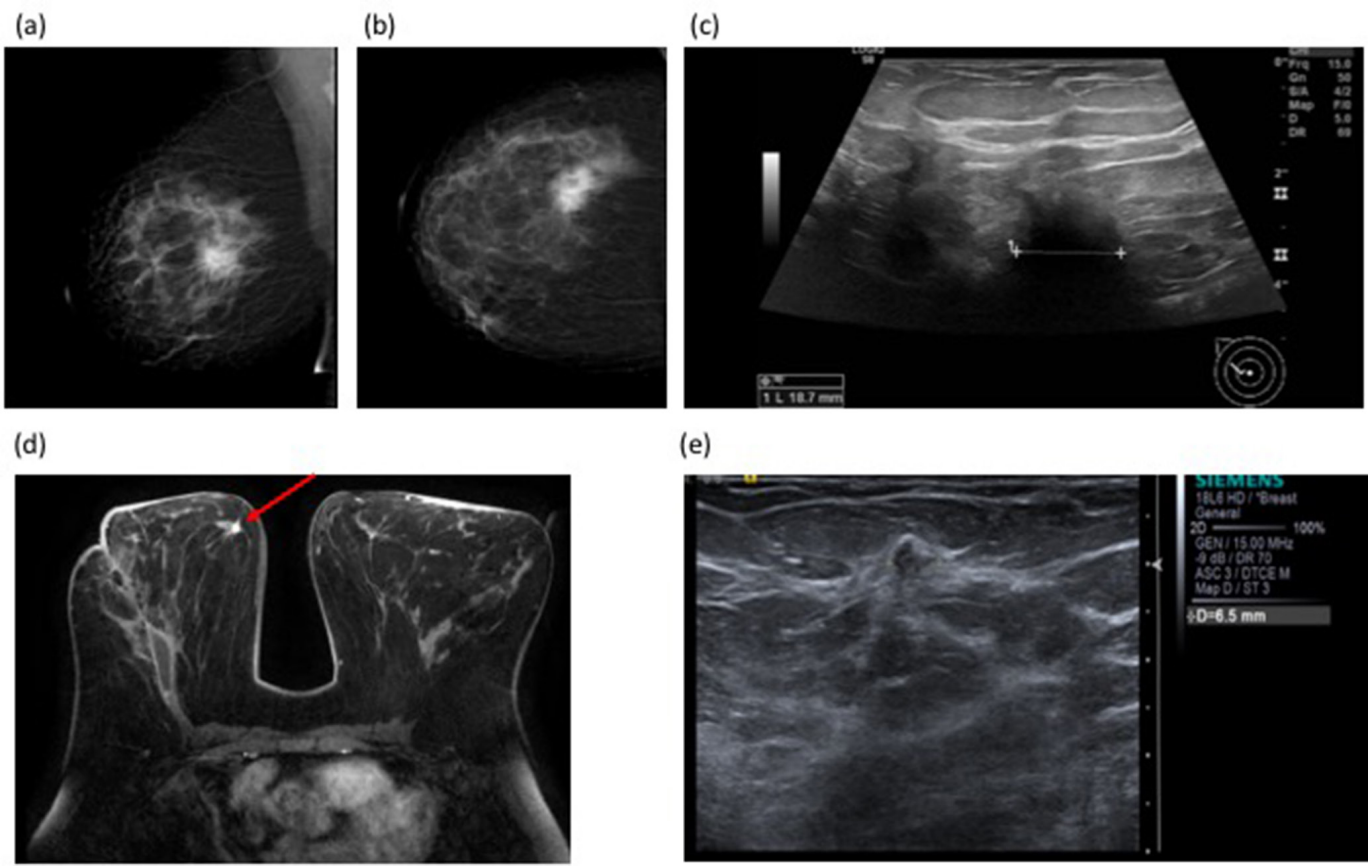
51-year-old female with a symptomatic right breast mass. Mammogram (BI-RADS b, scattered fibroglandular density) showed a 35 mm spiculate mass in the right upper outer quadrant Figure 3a and (b (Figure 2a and b), measuring up to 19 mm on ultrasound (Figure 2c) Figure 3c 14G core biopsy confirmed Grade 2 invasive NST. BCS was performed and histology showed a 60 mm Grade 3 NST with adjacent satellite nodules. The discrepancy in size between imaging and histology prompted post-operative breast MRI which demonstrated a 9 mm irregular enhancing mass in the right medial breast, distant from the site of surgery Figure 3d(Figure 3d). Second look ultrasound showed a corresponding 7 mm ill-defined mass in the medial right breast (Figure 3e). Figure 3e Core needle biopsy showed high-grade DCIS, and the patient proceeded to completion mastectomy. Final histology confirmed high-grade DCIS measuring 14 mm without invasive malignancy. BCS, breast conserving surgery;

Figure 2 illustrates a case where MRI prompted by discrepancy in size between initial imaging and WLE histology, revealed only expected inflammatory changes at the cavity but unsuspected high-grade DCIS distant from the surgical site altering management to completion mastectomy.

### Breast MRI abnormality surgical cavity only

Abnormal enhancement was confined to the surgical cavity with normal appearances on MRI in the remainder of the ipsilateral breast in 13 breasts (28%). One case with extensive residual pleomorphic LCIS at three of the resection margins went straight to mastectomy. 10 cases underwent re-excision as a second surgical procedure with one needing subsequent mastectomy. The remaining two patients in this group did not undergo any further surgery. In one, a small focus of low-grade DCIS was detected at the lateral margin. Breast MRI was concerning for disease at the surgical cavity and MDT decision was to accept the margins with a repeat breast MRI in 6 months, which proved normal. In the second case, a small focus of HG DCIS was present at the anterior margin and a small focus of DCIS in a lateral shave. The margins were accepted at MDT. Both of these patients underwent breast radiotherapy.

### Breast MRI abnormality at surgical cavity and also distant to surgical cavity

Abnormal enhancement was observed at both the surgical cavity and distant from this in seven breasts (15%) (Figure 1). Five of these proceeded directly to mastectomy whilst two underwent re-excision, one of which had persistently positive margins that mandated mastectomy. Therefore, a total of six mastectomies were performed in this group. For two of these seven cases, there was also concerning enhancement in the contralateral breast, as described above.

**Figure 3.**
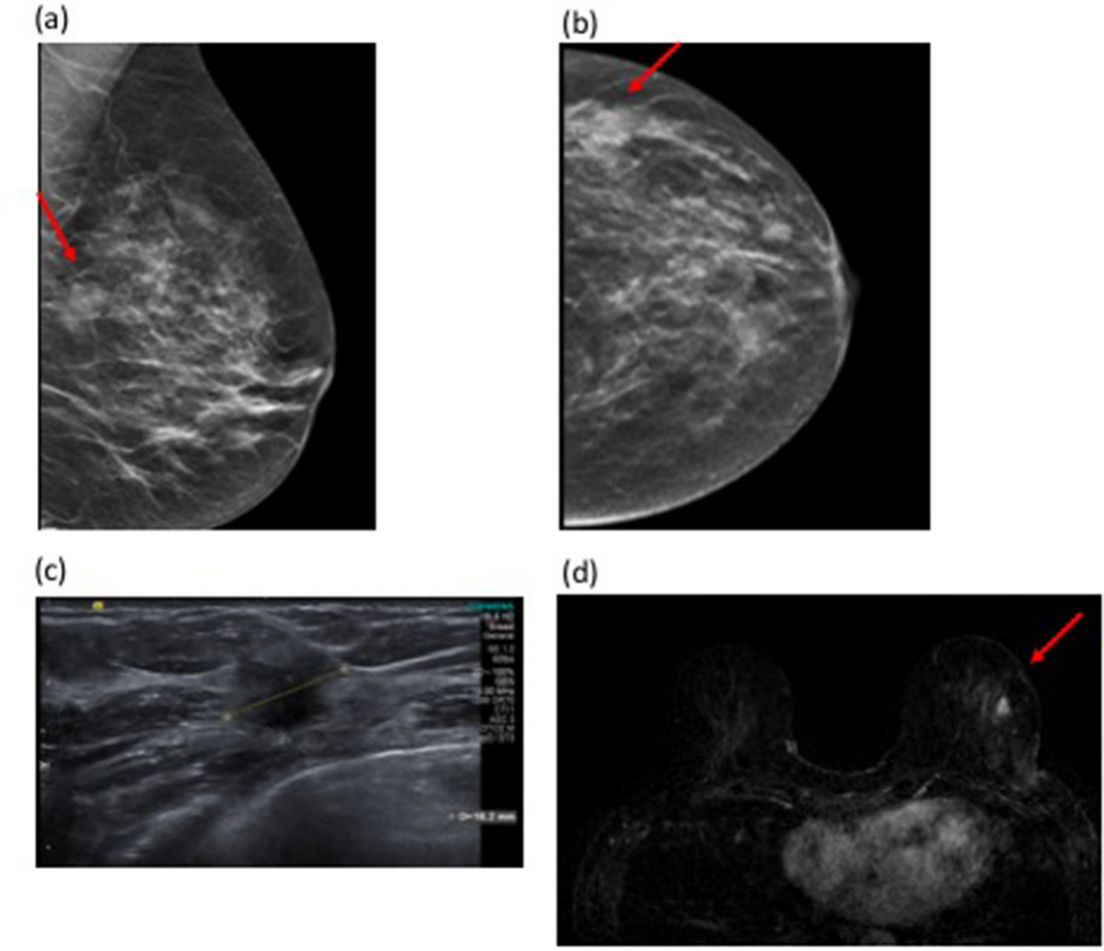
48-year-old female with a left breast lump and skin dimpling. Tomosynthesis shows an 18 mm mass in the lateral left breast (Figure 3a and b). Figure 3a and (bcorrelating with the clinical abnormality in a BI-RADS (c) (heterogeneously dense) breast. Ultrasound confirmed a hypoechoic solid mass suspicious for malignancy (Figure 3c). Ultrasound-guided 14G biopsy demonstrated Grade 2 invasive carcinoma (NST). Histology of the WLE specimen showed tumour involvement of the superior and medial margins and re-excision revealed persistent tumour involvement of the new resection margins. At this point, MRI was performed. MRI showed a 9 mm enhancing nodule at the site of surgery, suspicious for residual disease. A second lesion was seen remote from the surgical resection cavity in the lower outer quadrant, measuring 11 mm (Figure 3d). Second look ultrasound was normal, so MRI-guided biopsy was performed which confirmed invasive carcinoma with a similar histology profile to the original tumour. Completion mastectomy confirmed distant disease in the ipsilateral breast away from the surgical site. WLE, wide local excision.

Figure 3 demonstrates a case where MRI revealed residual disease at the surgical cavity and further invasive disease remotely, both confirmed at mastectomy.

### Changes in patient management

Post-operative breast MRI altered immediate patient management from re-excision surgery to an alternative in 21 cases (45%). The next steps for individual patient management are illustrated in Table 3 and ranged from needle biopsy to mastectomy. Although there was suspicious ipsilateral enhancement distant to the surgical cavity in 13 breasts, biopsy was performed in only 6 cases. These revealed invasive tumour of no special type (NST) in two cases, high-grade DCIS in a further two cases, and single B3 (LCIS) and B2 (benign) lesions. In those cases where breast biopsy for distant enhancing areas was not performed, three patients opted for mastectomy, two had benign correlates on second look ultrasound (lymph node and fat necrosis), and one had re-excision followed by mastectomy. In the seventh case, the MRI abnormality was located <20 mm from the surgical cavity and included in the re-excision surgery which was performed post-chemotherapy, with no residual invasive disease or DCIS on final histology. A total of 10 women (21%) underwent adjuvant chemotherapy prior to completion surgery and 13 mastectomies (28%) were ultimately performed.

**Table 3.** Impact of post-operative breast MRI on next step in patient management

**MRI changed next procedure from re-excision of margins to:**	**Number of cases** (***N* = 47**)
**Mastectomy**	5
**Chemotherapy**	7
**Biopsy of additional lesion ipsilateral breast**	6
**Biopsy of contralateral lesion**	3
**Total (%)**	21 (45%)

**Figure 4.**
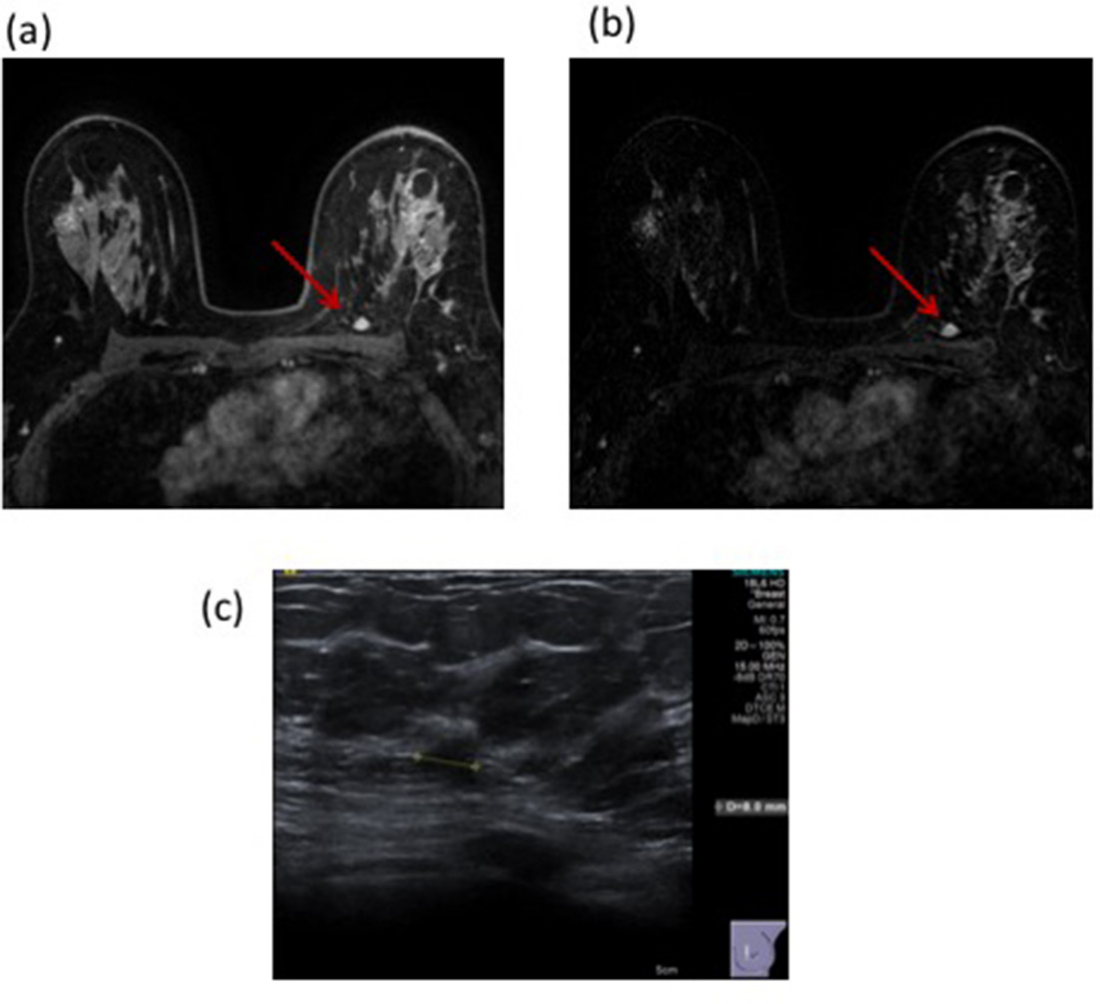
53-year-old female with a left breast mass. Mammography with density BI-RADS (b) demonstrated a 16 mm mass with a corresponding 22 mm solid mass on ultrasound. 14G core biopsy confirmed invasive NST. Following BCS, histology confirmed these findings but with involvement of the inferior margin. A post-operative MRI revealed suspicious enhancement at the surgical resection site with a further 10 mm area of enhancement posteriorly on both T1 non-subtracted (Figure 4a) and T1 subtracted post-contrast images (Figure 4b). This demonstrated Type II enhancement characteristics. Second look ultrasound detected a corresponding 8 mm indeterminate hypoechoic lesion (Figure 4c). Core biopsy revealed normal breast tissue only (**B1**). The patient underwent re-excision of the surgical cavity (all margins) but had no residual disease and has no evidence of recurrence after 4 years of follow-up with annual mammography. BCS, breast conserving surgery.

Figure 4 illustrates a case where MRI interpretation gave false-positive findings at both cavity site and remotely.

## Discussion

Pre-operative staging breast MRI is of proven value when employed for specific indications and is endorsed by official guidelines.^14–16^ Pre-operative MRI cannot however be justified routinely for all BCS patients for multiple reasons including resource constraints and pragmatism. In consequence, there will be a small number of females without pre-operative MRI who are found to have positive margins after BCS. What is the role of post-operative MRI in this situation?

The use of breast MRI in the early post-operative period is challenging due to enhancement at the surgical site which confounds interpretation and causes difficulty in confidently excluding residual disease at the margins of the cavity.^12,13,17,18^ This retrospective study shows that post-operative breast MRI is an infrequently performed investigation, comprising 2% of breast MRIs carried out at the authors’ institution and in 3% of patients who undergo BCS. The value of post-operative breast MRI is the provision of clinically useful information about the remainder of the breast and selection of patients for whom mastectomy is more appropriate as a next step rather than re-excision, as seen in 23% of our cases (11 of 47) following initial BCS. This study has shown that post-operative MRI changed surgical management from re-excision to mastectomy in almost one-quarter of cases.

Perhaps a more difficult decision is whether to re-operate or not for suspected local residual disease. Krammer et al determined a sensitivity and specificity of 72 and 73% respectively for detection of residual disease at the surgical resection cavity with post-operative breast MRI.^18^ In the same study, the sensitivity and specificity for pre-operative detection of multifocal or multicentric disease was reported as 90 and 96% respectively. Kim et al report a sensitivity and positive predictive value of post-operative breast MRI for detection of residual disease following BCS (51 cases) of 92.1 and 88.6%, but a correspondingly low specificity and negative predictive value of 69.2 and 56.3%.^19^ However, as for the current study, these other studies are limited by small numbers which precludes any robust conclusions.

When evaluating post-operative breast MRI for possible residual disease, it is important to combine assessment of morphologic and kinetic parameters. In a study of 207 cases, Young Chae et al, found that thick and irregular or nodular combined with non-mass like enhancement of the surgical resection cavity were characteristic of residual disease (sensitivity 80%, specificity 73%, positive-predictive value 87% and negative-predictive value 61%).^20^ Incorporation of both kinetics and morphological parameters increased specificity and positive-predictive value to 91 and 92% respectively. According to Kim et al the morphologic features of peripheral nodular enhancement, satellite nodule within 2 mm of cavity margin and irregular thickened cavity wall had a PPV of 83–100% for prediction of residual malignancy post BCS.^19^ With increasing use of post-operative MRI, radiological appearances will become more familiar, facilitating interpretation of images and permitting greater accuracy in evaluation.

There is much debate about the optimum timeframe for performing breast MRI in the post-operative setting. Stucky and colleagues report a decrease in sensitivity, specificity, PPV and NPV when breast MRI is performed more than 28 days after surgery compared with less than 28 days^21^. Frei and colleagues showed an increase in PPV from 69 to 92% when breast MRI was performed more than 28 days after initial surgery.^22^ However, both of these studies involved small numbers. In the current study, MRI was undertaken at a mean time of 62.5 days (range 13–112 days) following surgery.

MRI may also aid in further surgical management by detecting additional disease in the ipsi- or contralateral breast.^23,24^ However, in a study of 963 cases of DCIS, up to 28% had an abnormality on breast MRI that required further work-up/biopsy. In our study, in six cases additional biopsy was required, with three of these performed under MRI guidance. The requirement for additional biopsy, in particular MRI biopsy, adds to the cost of this already relatively expensive imaging tool. It will also pose a further physical and mental strain on patients who are uncertain if their treatment has been definitive.

There are a number of limitations to this study, including its retrospective nature and small case numbers. However, post-operative breast MRI is infrequently performed and this is a common limitation to other published studies.^12,19,21,22^ This study was observational with individualized indications for post-operative MRI within a pragmatic clinical context. Some patients with positive margins went on to receive radiotherapy alone or had chemotherapy prior to further surgery, thus making it difficult to the calculate true sensitivity and specificity of breast MRI. Whilst the commonest indication for post-operative MRI was positive margins, others included a search for occult residual disease where there was discrepancy between initial surgical histology and the pre-surgical work-up. Radiologists were not blinded to margin status at the time of reporting and this may have biased interpretation with a lower threshold for reporting residual cavity disease. However, it is routine practice for all MRIs to be double reported in our unit, and discussed at a breast MDT with consensus amongst a number of radiologists. Over the time period of the study, we have increased our capacity for performing breast MRI and are therefore now more likely to offer to perform pre-operative breast MRI in females with dense breasts and females with HG DCIS.

There is an increasing trend within the authors’ unit for surgeons to request post-operative MRI in females with positive margins after initial BCS, prior to considering additional surgical resection. This will impact on patient management and decisions for further surgery. We have shown that the value of post-operative MRI is in evaluation of unsuspected additional disease away from the surgical site, rather than in exclusion of residual tumour at the surgical resection cavity. In many cases post-operative breast MRI is requested to exclude disease at the surgical resection margin, which is challenging for radiologists to do definitively. This study has shown that MRI altered patient management from immediate re-excision to an alternative pathway in almost half (45%) of cases. The decision to perform post-operative breast MRI should not detract from the MDT plan to manage the potential residual disease. Detection of additional, distant disease on post-operative MRI will often prompt mastectomy rather than re-excision as a definitive next surgical procedure and avoid unnecessary multistep surgery or increased risk of future in breast recurrence.
